# A simple measure of the extent of Ebstein valve rotation with cardiovascular magnetic resonance gives a practical guide to feasibility of surgical cone reconstruction

**DOI:** 10.1186/s12968-019-0546-3

**Published:** 2019-06-27

**Authors:** Marina L. Hughes, Beatrice Bonello, Preeti Choudhary, Jan Marek, Victor Tsang

**Affiliations:** 1 Cardiorespiratory Unit, Great Ormond Street Hospital for Children NHS Foundation Trust, Great Ormond Street, London, WC1N 3JH UK; 2 Cardiology Department, Royal Papworth Hospital NHS Foundation Trust, Papworth Everard, Cambridge, CB23 3RE UK

**Keywords:** Ebstein anomaly, Tricuspid valve, Cone reconstruction, Congenital heart disease, Cardiovascular magnetic resonance imaging

## Abstract

**Background:**

Once surgical management is indicated, variation of Ebstein valve morphology affects surgical strategy. This study explored practical, easily measureable, cardiovascular magnetic resonance (CMR)-derived attributes that may contribute to the complexity and risk of cone reconstruction.

**Methods:**

A retrospective assessment was performed of Ebstein anomaly patients older than 12 years age, with pre-operative CMR, undergoing cone surgical reconstruction by one surgeon. In addition to clinical data, the CMR-derived Ebstein valve rotation angle (EVRA), area ratios of chamber size, indexed functional RV (RVEDVi) and left ventricular (LV) volumes, tricuspid valve regurgitant fraction (TR%) and other valve attributes were related to early surgical outcome; including death, significant residual TR% or breakdown of repair.

**Results:**

Of 26 operated patients older than 12 years age, since program start, 20 had pre-op CMR and underwent surgery at median (range) age 20 (14–57) years. TR% was improved in all patients. Four of the 20 CMR patients (20%) experienced early surgical dehiscence of the paravalve tissue, with cone-shaped tricuspid valve intact; one of whom died.

A larger EVRA correlated with Carpentier category and was significantly related to dehiscence. If EVRA >60^o^, relative risk of dehiscence was 3.2 (CI 1.3–4.9, *p* = 0.03). Those with dehiscence had thickened, more tethered anterior leaflet edges (RR 17, CI 3–100, *p* < 0.01), smaller pre-operative functional RVEDVi; (132 vs 177 mL/m2, *p* = 0.04), and were older (median 38 vs 19 years, *p* = 0.01). TR %, chamber area ratios and LV parameters were not different.

**Conclusions:**

Comprehensive CMR assessment characterizes patients prior to cone surgical reconstruction of Ebstein anomaly. Pragmatic observation of larger EVRA, smaller RVEDVi and leaflet thickening, suggests risk of repair tension and dehiscence, and may require specific modification of cone surgical technique, such as leaflet augmentation.

**Electronic supplementary material:**

The online version of this article (10.1186/s12968-019-0546-3) contains supplementary material, which is available to authorized users.

## Background

Ebstein anomaly of the tricuspid valve involves failure of delamination of the septal leaflet, and to a variable extent, failure of delamination of the inferior and anterior leaflets [1].

However, the anomaly shows remarkable morphologic and physiologic heterogeneity. It is strongly associated with right ventricular myocardial disease, conduction tissue anomalies and left ventricular (LV) noncompaction [2].

Cone reconstruction of the Ebsteinoid tricuspid valve is a complex surgery that aims to restore the anatomic position of the valve, with improved tricuspid valve competency through native, leaflet-leaflet apposition [3]. Although there are many subtle technical differences for each patient, this operation involves a series of procedures: extensive delamination of the tricuspid valve leaflets, closure of multiple fenestrations, fashioning a cone-shaped valve predominantly from the abundant anterior leaflet and the remnants of the septal and inferior leaflets, a reduction posterior commissuroplasty, a limited right ventricular plication over the diaphragmatic surface if indicated, and a partial ring or deVega annuloplasty, to support the repair.

The success of da Silva’s surgery [4] and subsequently that of other groups [5–7] has led to increasing uptake of this procedure in many centres, worldwide.

With critical perusal of results in our centre over time, we noticed that the cone reconstruction was not successful in the early post-operative period, in a minority of patients. Although tricuspid valve regurgitation had been effectively improved, some adult patients experienced early surgical dehiscence and breakdown of the repair, within 30 days of surgery.

Optimal case selection and patient-specific planning of surgical technique for this valve reconstruction appear to be crucial factors for success.

The current classification systems and recent data regarding Ebsteinoid valves are useful for gauging severity of disease, [8–11] but these systems and data do not always incorporate the variations of anatomy and haemodynamics that affect surgical methodology for cone reconstruction.

Although echocardiography is optimal for viewing valve leaflets and visualizing flow jets, cardiovascular magnetic resonance (CMR) can better characterize chamber volumes, 3-dimensional shapes and overall haemodynamics. CMR is not hampered by acoustic difficulties, and imaging views may be more easily standardized.

However, characterizing the variability and complexity of Ebstein anomaly requires images with high temporal and spatial resolution. CMR scan acquisition time is prolonged. Moreover, post-processing for volume segmentation of the right ventricle (RV) uses significant time resource. The technique is reproducible in experienced hands [12], but can be poorly reproducible for inexperienced teams. Therefore, the primary aim of our investigation was to prove that, rather than concentrating on laborious, potentially error-prone, volumetric analysis, CMR could provide additional rapid and pragmatic methods of assessing and measuring the Ebsteinoid tricuspid valve. This study specifically assesses the relationship between easily-measured CMR morphologic features and the early outcome of cone reconstruction. Our aim was to optimize the efficiency and accuracy of case selection, by identifying easily measureable features that may constrain effective cone reconstruction or modify the surgical techniques required. If these morphologic features could be more simply quantified and compared by the many units developing this specific surgical technique, then surgical planning and methodology for individual patients could be improved.

## Methods

### Design and patient selection

This was a retrospective analysis of all patients older than 12 years, undergoing the cone reconstruction for Ebstein anomaly in our combined adult and paediatric centres. A single surgeon completed all the operations from 2009, up to the present date.

This age-group selection was chosen because Ebstein patients needing intervention in infancy and early childhood more often have physiology that requires different strategies to those needing their initial intervention later in life. Many of the patients undergoing cone reconstruction younger than 12 years required a cavopulmonary anastomosis to supplement pulmonary blood flow. For standardization and consistency, we elected to exclude all younger patients to avoid bias due to extreme or surgically-altered physiology.

Patients were also excluded from this analysis if their tricuspid valve was dysplastic in a manner that did not fit diagnostic criteria for Ebstein anomaly, if previous attempt at surgical tricuspid valve repair had been undertaken, or if they had not undergone routine pre-operative CMR assessment.

Alternative Ebstein valve repair methods or primary tricuspid valve replacement was not undertaken in our centre during the surgical era of this cohort.

### Indications for surgery

The indications for cone reconstruction surgery were conventional, and were agreed at Unit multi-disciplinary team meetings. These included severe tricuspid valve regurgitation, progressive right atrial (RA) or RV dilatation, cyanosis, or development of symptoms of breathlessness or exertional intolerance [13]. No other method of Ebstein valve repair was undertaken in our center during the era of surgery of this studied cohort.

### Consent

Informed, signed consent for the use of anonymised CMR imaging data was obtained from all patients, or guardians of patients who were younger than18 years. The study protocol conforms to the ethical guidelines of the 1975 Declaration of Helsinki (National Research Ethics Service ethical code: 06/Q0508/124) (GOSH R&D no 07CC02).

### Cardiovascular magnetic resonance imaging methods

Scanning was performed for patients without sedation, using a 1.5 Tesla CMR scanner (Avanto, Siemens Healthineers, Erlangen, Germany). Anatomy was initially mapped using single-shot balanced steady state free precession (bSSFP) scout images in 3 radiological planes. Retrospectively gated, bSSFP cine images of the heart were planned and acquired in multiple planes, with each cine image acquired during a single breath-holding episode. The image planes included the vertical long axis of both LV and RV, the 4-chamber view, the LV and RV outflow tracts and the short axis; covering the entirety of both ventricles (9–12 slices). Additional transaxial cine images were acquired, in order to view the RV and tricuspid valve (TV) apparatus using at least 4 slices, evenly spaced in the transaxial plane between the level of the inferior (diaphragmatic) RV wall and the superior RV outflow tract.

**Fig. 1:**
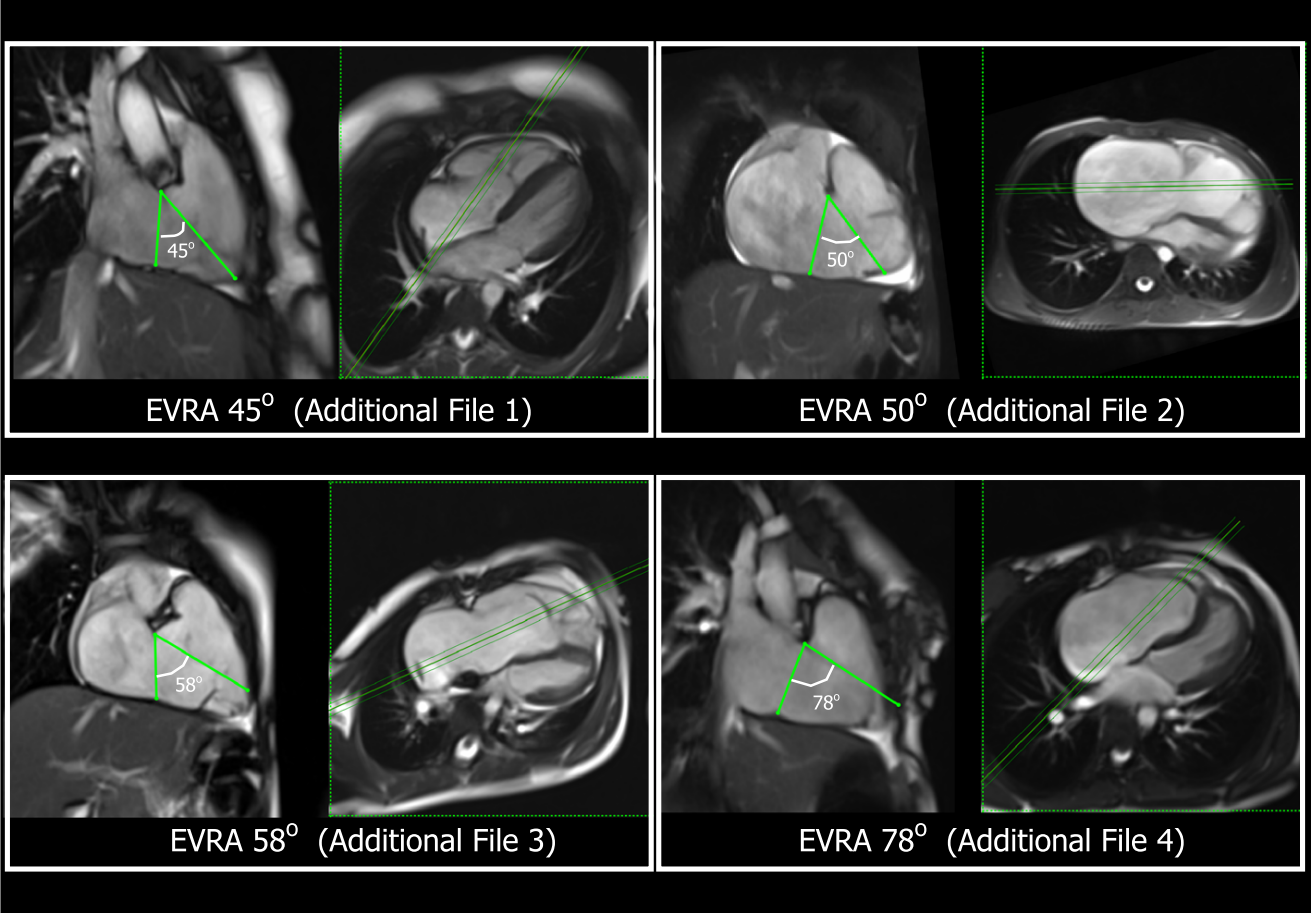
These panels illustrate the method of Ebstein valve rotation angle (EVRA) measurement in 4 different patients, using the end-systolic frame from cardiovascular magnetic resonance (CMR) balanced steady state free precession (bSSFP) cine images. In each panel, the left-hand image is the right ventricular (RV) long axis cine view, from which the EVRA is calculated. The right-hand image in each panel is the 4-chamber cine view from the same patient. The green triple-line demonstrates the position of the RV long axis view relative to the 4-chamber view, which is a perpendicular plane through the centre of the right atrio-ventricular (AV) junction, parallel to the interventricular septum. The EVRA for each of these patients is shown in green, drawn with apex at the superior basal hingepoint of the TV anterior leaflet in the RV long axis cine view. Each of these image panels corresponds to Additional movie files 1–4 in the supplementary data

The vertical long axis view of the RV (from which the Ebstein valve rotation angle (EVRA) was calculated) was planned from a 4-chamber or transaxial RV view, by placing a perpendicular slice passing through the centre of the native, right atrio-ventricular junction, parallel to the interventricular septum (Fig. 1).

The bSSFP cine imaging was acquired using the following parameters: repetition time (TR) 42.6 ms, echo time (TE) 1.2 ms; flip angle 68°, slice thickness 6-10 mm, matrix 200 × 240, field of view 300-380 mm, and temporal resolution 20 true phases per cardiac cycle, acquired during a single breath-holding episode.

Through-plane CMR phase-contrast flow quantification data were acquired at four positions: the ascending aorta, the main pulmonary trunk and in each branch pulmonary artery, proximal to the first branch. The flow-imaging plane was planned truly perpendicular to the vessel and acquired during breath-hold, using a velocity-encoded, retrospectively gated, spiral phase contrast CMR sequence [14]. Scanner settings for the flow measurements were as follows: spatial resolution 1.6 × 1.6 × 5 mm^3^, TE/TR 2.08/32.0, flip angle 25°, encoding velocity 180-300 cm/s, 20 phases.

### Post processing

All images were processed using an in-house plug-in for the open source DICOM software OsiriX (OsiriX Foundation, Geneva, Switzerland) [15]. However, similar post-processing tools are available on other commercial software reporting platforms for CMR.

All of the post-processing was completed in a standardized manner, by experienced CMR reporting staff, with more than 10 years experience. Volume and flow measurements were indexed for body surface area and expressed in mL/m2.

Assessment of LV and RV volumes was performed by manually tracing the endocardial borders of both ventricles at end-diastole and end-systole, using the short axis stack of cine images. Myocardial trabeculations and larger papillary muscles were excluded from the blood pool. The atrialised RV volume (on the atrial side of any visible TV leaflet apparatus) was not differentiated from true RA volume, but was differentiated and excluded from the true, functional RV volume, by carefully tracing the border of the functional TV tissue. Therefore, the RV end-diastolic volume index (RVEDVI) refers to the body surface area (BSA)-indexed functional RV volume. This study did not attempt to segment and differentiate the RA chamber from the “atrialised” RV chamber, as this has the potential for significant subjectivity.

During segmentation, all available long axis cine planes were used as reference indicators for accurate slice position. This segmentation allowed evaluation of biventricular end-diastolic volume (EDV) and end-systolic volume (ESV), and calculation of stroke volume (SV) and ejection fraction (EF) [12].

Aortic and pulmonary flow volumes were measured from the phase contrast CMR data. This was segmented using a semi-automatic vessel edge detection algorithm with manual operator correction (OsiriX plugin).

The tricuspid valve regurgitant fraction (TVRF) percent was calculated using the RV stroke volume and pulmonary artery (PA) forward flow volume, using the following equation: TVRF % = [(RVSV – PA forward flow) / RVSV] × 100.

**Fig. 2:**
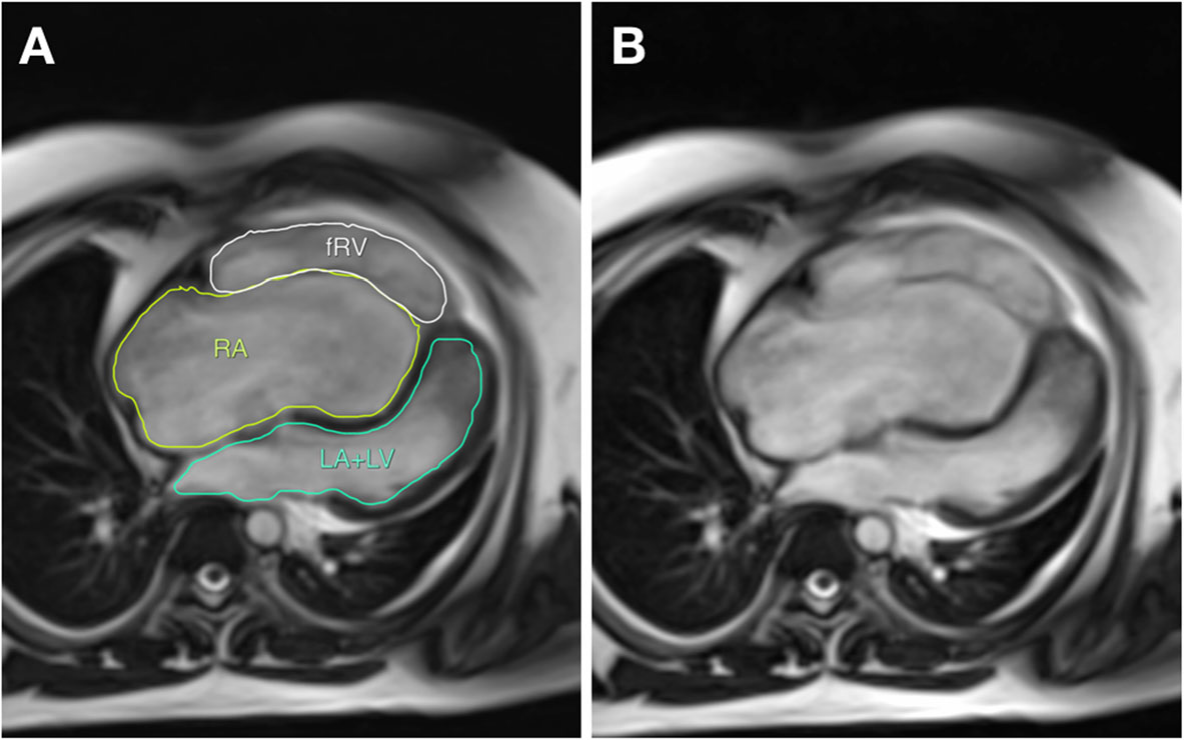
This is an end-diastolic frame from a 4-chamber view, using the CMR bSSFP sequence. The left-hand panel (**a**) shows the segmentation and tracing method for area ratios of the right and left heart, superimposed upon the image shown on the right, without segmentation (**b**). fRV = functional RV, RA = right atrium, LA + LV = left atrium + left ventricle

The (right atrium: rest of heart) area ratio was measured from the 4-chamber CMR image, using the principles of Celermajer et al. [9]. Additionally, a simplified, area-based version of the volume ratio proposed Hosch et al. [10] was calculated, using the end-diastolic frame from the 4-chamber cine view. The RA border was traced in detail around the atrial endocardium and along the leaflet margin of the tricuspid valve as shown in Fig. 2. The functional RV (fRV), LV and LA endocardial areas were similarly traced. Left heart area included (LA + LV) area, and “rest of heart area” included (fRV + LA + LV) area.

### The Ebstein valve rotation angle EVRA

The EVRA was defined as the plane of systolic apposition of the tricuspid valve leaflets, when viewed using a 2-dimensional RV long axis (RVLA) cine image (Fig. 1, and Additional files 1, 2, 3, 4). With Ebstein anomaly, the systolic valve apposition plane in the RVLA view is usually formed by components of the anterior leaflet alone, or between the anterior and partially-laminated inferior leaflet.

The end-systolic frame was selected from the cine loop, and the EVRA was measured with the fulcrum of the angle positioned at the basal superior “hinge-point” of the valve in this cine view, anterior to the aortic valve. This is reminiscent of the observations and illustration made by Schreiber et al. These authors describe the rotation of the valve within anatomic specimens, with the fulcrum at the ventriculo-infundibular fold [16].

The first axis of the angle was drawn from the inferior, right atrio-ventricular (coronary) groove, towards the fulcrum. The second axis was drawn from the fulcrum, through the systolic plane of valve coaptation, then towards the RV free wall (RV inferior wall, RV apex or anterior free wall). This systolic apposition plane was often best marked by the origin of the “vena contracta” of the TV regurgitant jet. The EVRA is the angle between these two axes. Figure 1 and Additional files 1, 2, 3, 4 demonstrate this angle in four different subjects from this cohort. With Ebstein anomaly, there are varying degrees of rotation of the systolic apposition plane towards the RV outflow tract [16]. A person with a normal TV would have no rotation of the plane of systolic closure, and therefore a rotation angle of 0 degrees.

The EVRA for each patient was measured twice, by each of two experienced, independent observers, who were blinded to each other’s and the surgical results at the time of analysis. Repeat measurements by both observers were undertaken after a 2–4 week interval.

### Surgical methods

The surgical technique is explained in the introduction of this manuscript and has been previously described in detail [6]. A partial ring or deVega annuloplasty (avoiding the conduction tissue) is routinely performed to support the reconstructed cone-shaped valve, except in neonates or small infants.

### Outcome criteria

The pre-defined short-term primary outcome measures were

- early death, within 30 days of surgery
- early dehiscence of the reconstructed valve apparatus, within 30 days of surgery
- severe residual tricuspid valve regurgitation

**Fig. 3:**
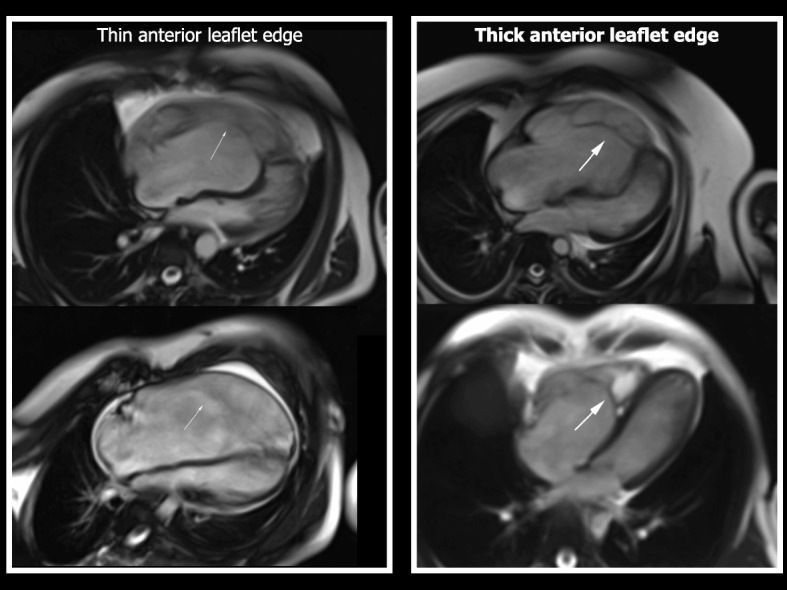
This figure shows end-diastolic frames from a 4-chamber view, using bSSFP images, demonstrating the morphologic characteristic of thickened edges of the anterior leaflet. Two patients with thickened anterior leaflet edges are shown on the right-sided panels (thick white arrows), compared to two patients in this surgical cohort with similar EVRA and RV size, with valves that did not exhibit thickened edges (thin white arrows) on cine images

The CMR factors assessed were: Carpentier classification [8], Ebstein valve rotation angle (EVRA), RA: rest of heart area ratio of cardiac chambers (from 4-chamber view) (see Fig. 2), BSA-indexed functional RV and LV end-diastolic volume (RVEDVi, LVEDVi), BSA-indexed functional RV and LV end-systolic volume (RVESVi, LVESVi) and TV regurgitant fraction (TR%). Additionally, cine images were observed to record the presence of thickening of the edges or leading margins of the TV anterior leaflet. Figure 3 illustrates a diastolic frame from bSSFP cine images from two patients in the cohort exhibiting thickening of the anterior leaflet edges, and two patients, with similar EVRA and RV size, who do not show obvious anterior leaflet edge thickening.

### Statistics

Categorical variables were expressed as proportions. Continuous variables were expressed as medians with full range.

Graphpad Prism 7.0c (GraphPad Software, La Jolla, California, USA) was used for statistical analysis and figures. Simple, non-parametric statistical tests compared the groups (Mann-Whitney U test, Kruskall-wallis test for continuous variables, Fisher’s exact test for categorical groups, and Spearman for correlation). Small numbers precluded valid use of multivariate regression methods. Bland-Altman methodology assessed inter-observer and intra-observer variability. The level of α considered for statistical significance was 0.05.

The authors had full access to the data and take responsibility for its integrity. All authors have read and agreed to the manuscript as written.

## Results

### Cohort description

Between 2009 and May 2018, a total of 26 patients older than 12 years had undergone cone reconstruction of the native Ebstein tricuspid valve in our unit, having met conventional criteria for surgery.

Six of these 26 patients were not examined with pre-operative CMR, and have been excluded from this analysis. These patients were operated primarily during the earlier years of this surgical program, and because of referral from other centres, had no authorization for CMR in addition to preoperative echocardiographic imaging. None of these 6 patients experienced a sub-optimal short-term surgical result. Therefore, with the established selection criteria, the cohort for this study included 20 patients (11 male).

### CMR examinations

The CMR examinations were undertaken at a median (range) age 19 (13–56) years, and the surgery was undertaken at an age of 20 (14–57) years. The median (range) between pre-operative CMR and surgery was 0.8 (0–4) years. For one adult the time between CMR and surgery was greater than 18 months, but the essential valve structure does not change, and echocardiography had shown no significant changes during that time period, so this CMR data was included in the study.

### Clinical description

Table 1 describes the pre-operative physical and cardiac parameters of the 20 studied patients. Additionally, 8 patients had a small atrial septal defect (ASD). Three of these had a right to left shunt through the defect, causing mild cyanosis at rest, with CMR-measured Qp:Qs between 0.8–0.9: 1. Three patients had a left to right shunt through the small ASD, giving CMR-measured Qp:Qs between 1 and 1.5: 1. Two patients had no measureable net shunt through the defect, under scanning conditions.

**Table 1:** Pre-operative parameters of the cohort, n = 20

Parameter	Median (range) or ratio
Gender (M:F)	11: 9
Age at surgery (years)	20 (14–57)
Body weight at surgery (kg)	68 (43–99)
Cardiothoracic ratio	0.66 (0.50–0.74)
RA area index (cm2/m2)	34.0 (10.4–54.9)
Right heart area (RA + fRV): Left heart area (LA + LV) ratio	2.7 (1.1–7.3)
RA area:Rest of heart area ratio [RA:(fRV + LA + LV)]	0.96 (0.29–1.80)
Functional RV end-diastolic volume index (mL/m2)	169 (83–353)
Functional RV end-systolic volume index (mL/m2)	93 (47–209)
LV end-diastolic volume index (mL/m2)	49 (34–74)
RVEDVi: LVEDVi ratio	3.2 (1.6–8.8)
RV ejection fraction (%)	46 (26–60)
LV ejection fraction (%)	61 (44–74)
Tricuspid valve regurgitant fraction (%)	55 (24–85)
Ebstein valve rotation angle (EVRA) (degrees)	54 (20–78)

*EDVi* end-diastolic volume index, *fRV* functional right ventricle, *LA* left atrium, *LV* left ventricle, *RA* right atrium, *RV* right ventricle

Of the whole group, the median (range) body weight at surgery was 68 (43–99) kg.

### Carpentier classification of the TV and EVRA

Using imaging and surgical report, 6 patients were classified as Carpentier Type A, 9 Carpentier Type B and 5 Carpentier Type C [8].

**Fig. 4:**
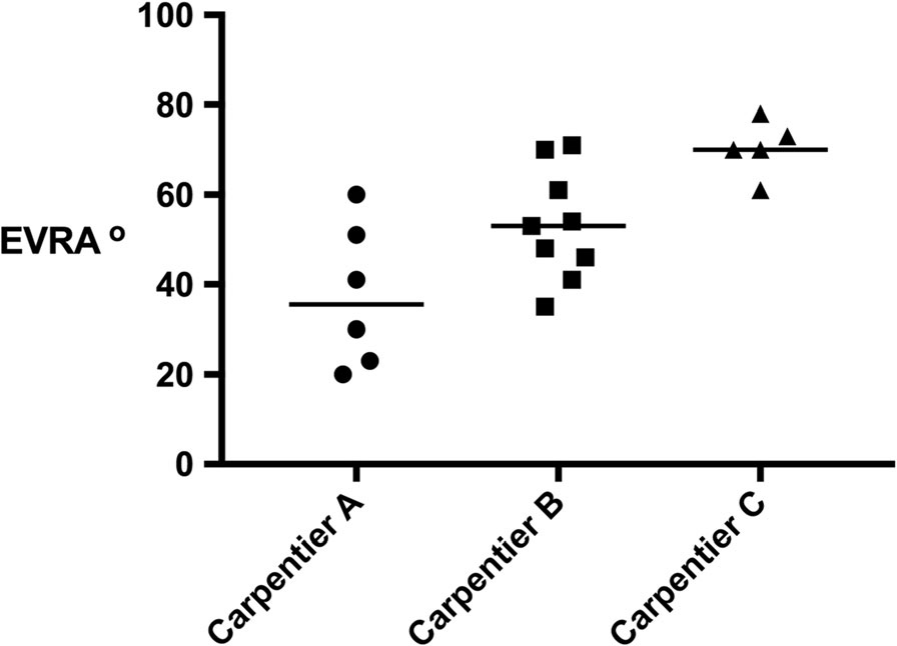
Scattergram of the measured Ebstein valve rotation angle (EVRA) for the whole cohort, *n* = 20, stratified by Carpentier classification

The EVRA was measureable in all patients. For all patients, the measured EVRA correlated with the Carpentier classification of valve anatomy, as classified by the imaging team and surgeon. However there is some overlap of EVRA between Carpentier grades. Illustrating this, a scatter diagram of the distribution of EVRA in the whole patient cohort according to Carpentier classification is given in Fig. 4.

Correlation of the EVRA with other quantitative CMR parameters, including functional RV end-diastolic volume, total RA area, RV EF and TV regurgitation was tested, but none were found significant in this limited cohort.

On assessment of cine images, three patients in this cohort exhibited prominent thickening of the anterior leaflet margin or edge. With thickened, rolled edges of the anterior leaflet, there seemed to be a greater number of leaflet attachments to the RV anterior wall, with less leaflet mobility (Fig. 3). The EVRA was significantly greater in patients with thickening of the anterior leaflet. In these three patients median (range) EVRA 73 ^o^ (70 ^o^ − 78 ^o^) vs 51 ^o^ (20 ^o^ − 71 ^o^) in the 17 patients without obvious thickening, *p* < 0.01.

Among patients with a small atrial septal defect (ASD), the EVRA was compared between patients with net right to left shunt, versus those with a net left to right shunt. The EVRA did not show a clear relationship with the presence of an ASD, or with the direction of shunt if a small ASD was present. Of the six patients with a small ASD, there was no difference of the EVRA in the three shunting right to left, compared to the EVRA of the three shunting left to right.

**Fig. 5:**
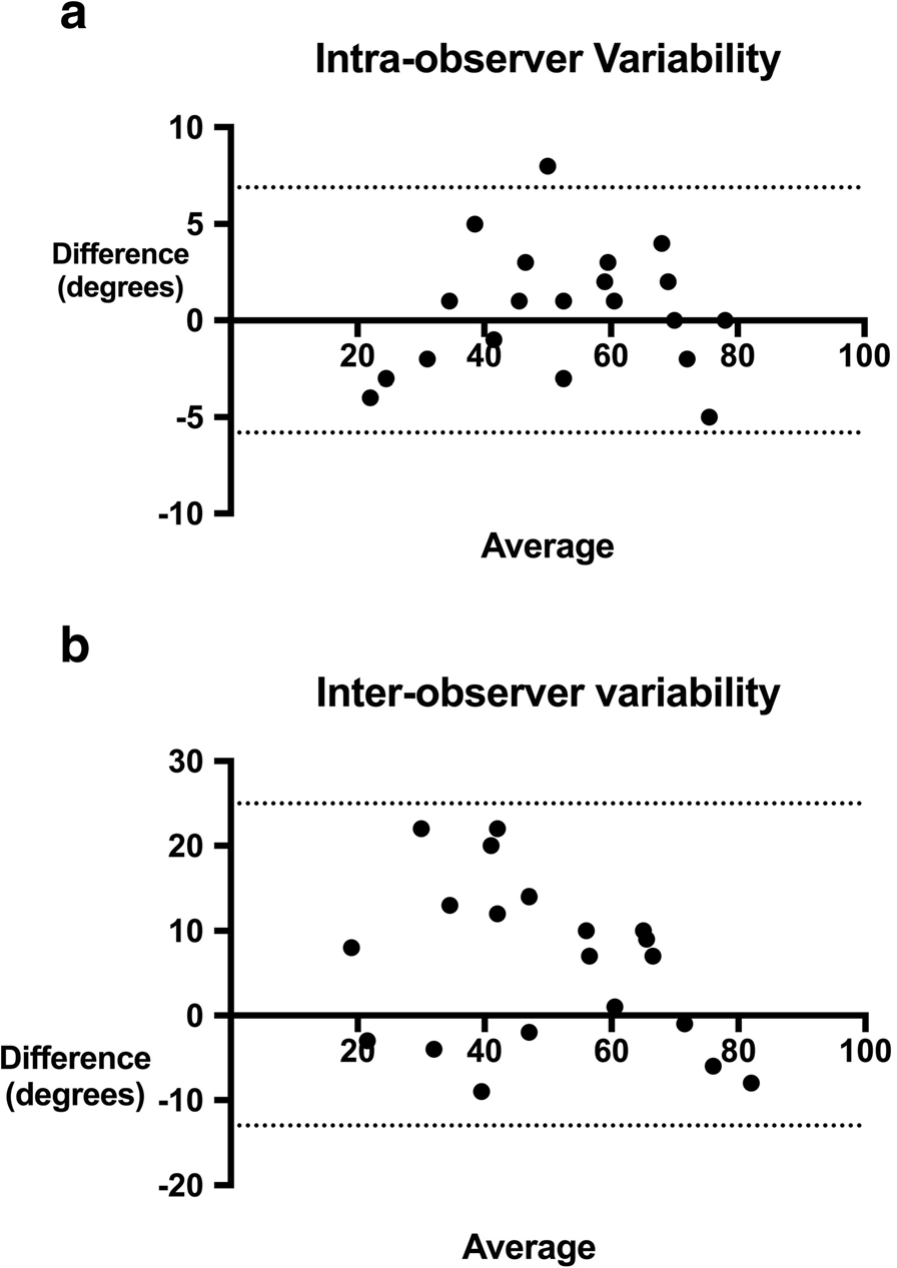
**a** and **b** Bland Altman plots showing intra and inter-observer variability measuring Ebstein valve rotation angle (EVRA). The dotted lines show the 95% Limits of Agreement

Using Bland Altman Methodology, we found reasonable agreement between experienced observers measuring the EVRA, (Fig. 5a and b). Figure 5a illustrates the intra-observer variability, measured in degrees, with maximum difference between measures <10^o^. The standard deviation was 3.2^o^ with 95% limits of agreement being − 6^o^ to + 7^o^.

The standard deviation of variability in EVRA between two experienced observers was 9.7^o^, with 95% limits of agreement being − 13^o^ to + 25^o^. Therefore, observers tended to differ in angle measurements by up to 25 ^o^ degrees. The difference between observers was greatest when angle measurements were at the lower range of values; that is, less rotated valves.

### Patient outcomes

TV regurgitation was improved in all patients. Using echocardiography, the early post-operative grade of residual regurgitation through the reconstructed valve apparatus was recorded as mild or moderate in all patients.

No patients in this cohort (> 12 years of age) underwent bidirectional cavopulmonary anastomosis.

Four of the 20 patients experienced early, para-valve surgical break down of the cone reconstruction, all within 30 days of surgery. These tended to occur more during earlier years of the 2009–2018 surgical program, but with no measureable significant clustering. The serial surgical case number and year of surgery for each of these four patients was case 6 (2010), case 10 (2013), case 14 (2014), case 16 (2014). One of these four patients subsequently died, 6 days post-operatively, with right heart failure and multi-organ dysfunction.

The mechanism of repair breakdown in each patient was tension and subsequent dehiscence caused by detachment of the septal margin of the surgical repair, leaving the cone-valve apparatus intact, but allowing a significant “paravalvar” leak. In one patient the posterior commissuroplasty sutures dehisced, and was subsequently repaired with a bovine pericardial patch. In two other patients the septal component of the cone repair dehisced. One of these patients has undergone re-repair, with bovine pericardial patch to repair the dehisced section. All patients undergoing re-repair subsequently had an uneventful recovery. No patient has undergone TV replacement.

Table 2 portrays the comparison of CMR parameters between the patients suffering early surgical breakdown of repair, with those in whom cone repair remained intact. Patients suffering dehiscence were older than those with intact repair (median 38 years (33–57) vs 19 years (14–45), *p* = 0.01).

**Table 2:** Comparison of patients with early surgical breakdown of cone reconstruction, versus those with intact repair

Parameter	Surgical dehiscence (*n* = 4)	Intact cone reconstruction (*n* = 16)	
Ebstein valve rotation angle (EVRA) (degrees)	72 (60–78)	50 (20–71)	*p = 0.01
Functional RV end-diastolic volume index (mL/m2)	132 (83–168)	177 (101–353)	*p = 0.04
Age at surgery (years)	38 (33–57)	19 (14–45)	*p = 0.01
Gender (M:F)	3: 1	8: 8	*p* = 0.59
Cardiothoracic ratio	0.63 (0.55–0.68)	0.68 (0.5–0.74)	*p* = 0.24
RA area index (cm2/m2)	32 (25–36)	34 (10–55)	*p* = 0.62
R heart (RA + fRV): L heart (LA + LV) area ratio	1.9 (1.4–3.2)	2.7 (1.1–7.3)	*p* = 0.17
RA area: Rest of heart area ratio [RA:(fRV + LA + LV)]	0.94 (0.77–1.2)	0.96 (0.29–1.8)	*p* = 0.96
LV end-diastolic volume index (mL/m2)	50 (34–63)	49 (34–74)	*p* = 0.87
RVEDVi: LVEDVi ratio	2.4 (1.6–4.9)	3.6 (1.6–8.8)	*p* = 0.22
RV ejection fraction (%)	46 (38–47)	44 (26–60)	*p* = 0.99
LV ejection fraction (%)	57 (53–68)	63 (44–74)	*p* = 0.35
Tricuspid valve regurgitant fraction (%)	43 (30–75)	67 (30–85)	*p* = 0.40

Legend of terms for Tables 1 and 2

*RV* right ventricle, *LV* left ventricle, *RA* right atrium, *LA* left atrium, *fRV* functional right ventricle, *RVEDVi* RV end-diastolic volume index, *LVEDVi* LV end-diastolic volume index

All results shown as median (range)

The pre-operative functional RV volume (RVEDVi) was smaller in those patients with breakdown of surgical repair, (median 132 mL/m2 (83–168) vs 177 mL/m2 (101–353), *p* = 0.04).

**Fig. 6:**
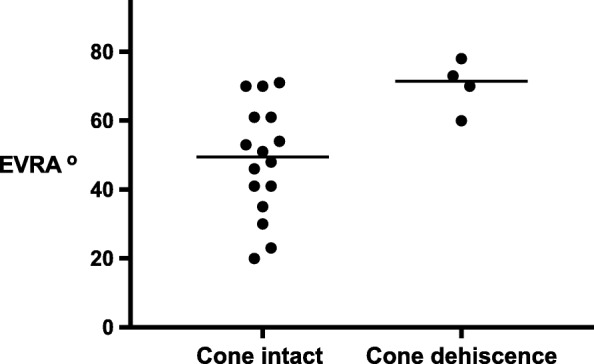
Scattergram of the measured Ebstein valve rotation angle (EVRA), comparing patients with early surgical dehiscence of the cone reconstruction (*n* = 4), compared with patients with an intact repair. (*p* = .0.01). This indicates that the effective tricuspid valve orifice and plane of systolic closure was more rotated towards the RV outflow tract in the patients suffering early surgical dehiscence

Associated with this, and simpler to measure, the Ebstein valve rotational angle (EVRA) was significantly greater in the 4 patients suffering surgical dehiscence (EVRA 72 ^o^ (60–78) vs 50 ^o^ (20–71), p = 0.01). This is illustrated in the scatterplot, Fig. 6. This data indicates that the effective TV orifice and plane of systolic closure was more rotated towards the RV outflow tract in the patients suffering surgical dehiscence. If EVRA was greater than 60^o^, the relative risk of dehiscence was 3.2 (CI 1.3–4.9, *p* = 0.03).

Three of the 4 patients with surgical dehiscence exhibited thickened, rolled edges of the anterior leaflet, which as described previously, was noted to occur with numerous leaflet attachments to the RV anterior wall, and poor leaflet mobility. None of the 16 patients without dehiscence exhibited thickened, rolled anterior leaflet margins. For patients with a thickened anterior leaflet edge the relative risk of dehiscence in this cohort was 17, CI 3–100, *p* < 0.01.

Other CMR indicators of Ebstein disease severity: RVEDV: LVEDV ratio, RVEF, LVEF, TR%, cardiothoracic ratio, and the calculated area ratios, did not differentiate patients at risk of early surgical tension and breakdown in this surgical cohort.

The shunt at atrial level can also give indication of the physiology of the Ebsteinoid valve and RV. The presence of an atrial shunt, and the net direction of shunting did not show a relationship with surgical outcome in this cohort. Of the four patients with early surgical dehiscence, two had no ASD. Of the two who did have a small ASD, one net shunt was right to left, and the other was left to right.

## Discussion

Within the diagnostic definition of Ebstein anomaly, there is huge variation of valve and ventricular characteristics, haemodynamics and clinical symptoms. The main aim of this study was to use CMR to study patients who had been selected for surgery, and to identify practical, easily measurable attributes of the Ebsteinoid valve that may affect the surgical technique required, or the outcome of cone reconstruction. Ultimately, the aim is to improve case selection for this complex surgical repair, and to optimise the specific techniques used within the reconstructive process.

This study only involved patients who had been clinically justified for intervention, and referred for surgery. This study did not judge severity of disease, nor analyse characteristics for referral.

We identified that a simple measure of the angle of rotation of the coaptation plane of the tricuspid valve, (the EVRA) predicted surgical outcome in our cohort.

For most patients, the EVRA correlates loosely with a Carpentier-type classification, but the EVRA is a more pragmatic, quantitative parameter, easily measureable in every patient.

We suggest that measuring this angle may help to identify those Ebstein valves with less abundant leaflet tissue that is available for the cone reconstruction. The EVRA may indicate those cases where the tension in the reconstruction will be greater, and thus greater risk of surgical breakdown. The surgical strategy for those patients with a greater EVRA is highly likely to require augmentation of leaflet tissue to complete the valve repair.

As described previously [6] a partial ring annuloplasty is routinely performed to support the reconstructed cone-shaped valve. However the surgical dehiscence in our series was mainly related to detachment of the septal component of the valve. The mechanism is likely due to the high tension on the sutures that connect the mobilised, apically deviated valve tissue to the septal muscular tissue near the atrioventricular junction in the presence of a dilated right ventricle.

Of note, since identification of these dehiscence cases in our centre, the surgical technique has been recently modified. Pledgeted sutures are now routinely used in the posterior commissural area, and the septal components of the cone valve are augmented with pericardial tissue and extended to minimize tension, if judged necessary.

Greater rotation of the valve, with a larger EVRA, also correlated with a more tethered, fibrosed and thickened anterior valve leaflet. The variations and number of anterior leaflet attachments and fenestrations were not independently quantified in our study, but these issues are also a crucial aspect of the technical challenge for effective valve reconstruction, and could be quantified with detailed imaging.

The pre-operative functional RV end-diastolic volume was lower in the patients in whom cone reconstruction broke down early after surgery. Thus, a salient finding in our study is that the specific prognosis of surgical cone reconstruction does not necessarily align with the conventional clinical severity indices that may give natural prognosis of native Ebstein anomaly [9–11].

The EVRA was measureable in all patients, with reasonable reproducibility of the measurement. The difference between observers was greatest when angle measurements were at the lower range, which is less critical if using this angle as a surgical decision tool.

Limitations: Despite the clear findings, we acknowledge that the study methods have some limitations, inherent in any retrospective clinical analysis.

Although standardized surgical methodology was used, there is possible confounding due to an era effect, and evolution over time of the surgeon’s skill, experience and technique.

This study is not an analysis of disease severity with regard to surgical outcome, nor is it a study of the clinical selection criteria for surgical intervention. This study specifically assessed the effect of easily-measured CMR morphologic features on the early surgical outcome of cone reconstruction. Although there may have been subtle learning over time regarding case selection for surgery, the selection criteria for referring each patient for surgical intervention were not the focus of this investigation. In our Unit, patients were selected for surgery based on consensus among clinicians at multidisciplinary meetings, with the use of subjective and objective data involving CMR, echocardiography, exercise testing, and clinical symptoms.

## Conclusion

For Ebstein patients selected for surgical repair, comprehensive CMR assessment can characterize the valve and ventricles prior to cone reconstruction. Pragmatic observation of larger EVRA, smaller functional RVEDVi and leaflet edge thickening suggest risk of repair tension and dehiscence, and may demand specific modification of cone reconstruction technique. A larger EVRA may indicate need for leaflet augmentation.

Although prospective testing is now needed, we suggest that this assessment process could help to improve case-selection, guide surgical methodology and therefore help to optimise cone surgical outcome for this complex group of patients.

## Additional files

Additional file 1: Method of Ebstein valve rotation angle Patient 1 (EVRA 45 degrees). (MOV 6755 kb)

Additional file 2: Method of Ebstein valve rotation angle Patient 2 (EVRA 50 degrees). (MOV 4189 kb)

Additional file 3: Method of Ebstein valve rotation angle Patient 3 (EVRA 58 degrees). (MOV 6792 kb)

Additional file 4: Method of Ebstein valve rotation angle Patient 1 (EVRA 78 degrees). (MOV 6323 kb)

The four Additional files are movies corresponding to the image panels in Fig. 1. The movies illustrate the method of EVRA measurement using the end-systolic frame from CMR bSSFP cine images in four different patients from this cohort. In each file, the left-hand image is the RV long axis view, from which the EVRA is calculated. The EVRA is shown with green angle lines, in the end-systolic frame.

The right-hand image in each panel is the 4-chamber view from the same patient. The green triple-line demonstrates the position of the RV long axis view relative to the 4-chamber view; a perpendicular plane through the centre of the right AV junction, parallel to the interventricular septum.

## Data Availability

The datasets used and analysed during this study are available from the corresponding author on reasonable request.

## Abbreviations

ASD: Atrial septal defect
BSA: Body surface area
bSSFP: Balances steady state free precession
CMR: Cardiovascular magnetic resonance
EDV: End-diastolic volume
EF: Ejection fraction
ESV: End-systolic volume
EVRA: Ebstein valve rotation angle
fRV: Functional right ventricle
LA: Left atrium/left atrial
LV: Left ventricle/left ventricular
LVEDVi: Left ventricular end-diastolic volume index
PA: Pulmonary artery
RA: Right atrium/right atrial
RR: Relative risk
RV: Right ventricle/right ventricular
RVEDVi: Right ventricular end-diastolic volume index (functional RVEDVi)
RVLA: Right ventricular long axisTR% Tricuspid valve regurgitant fraction
TV: Tricuspid valve
